# Supplementary material for: Observation of Weyl nodes and Fermi arcs in tantalum phosphide
Source: Nat Commun. 2016 Mar 17;7:11006. doi: 10.1038/ncomms11006 (PMC4800437; doi:10.1038/ncomms11006)
Supplement: Supplementary Information — Supplementary Figures 1-5 and Supplementary Reference [file ncomms11006-s1.pdf]

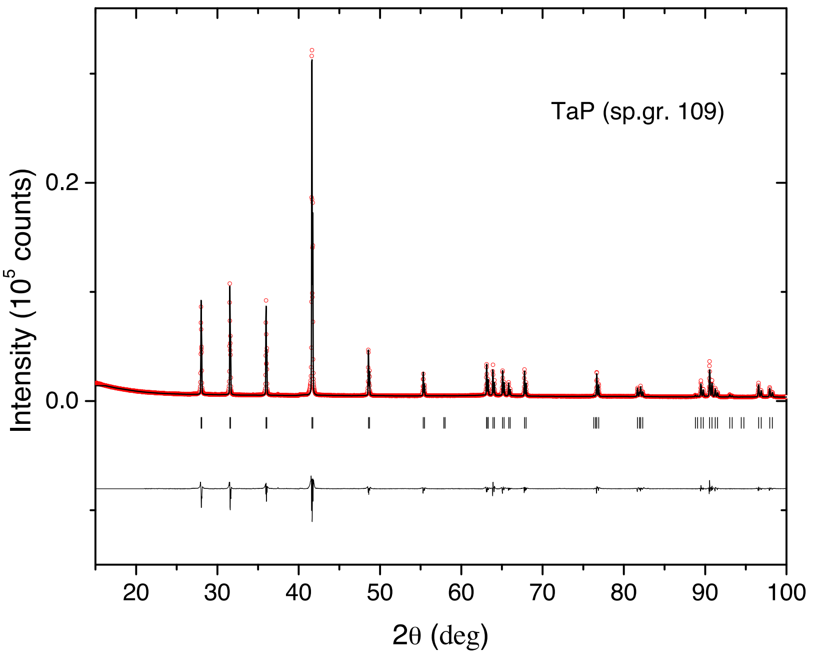

Supplementary Figure 1: X-ray diffraction data on TaP. The x-ray diffraction measurements have been performed on TaP powder at room temperature using a Brucker D8 diffractometer with Cu Ka radiation. Powder matching pattern (LeBali fit) (upper-red) and difference plot (lower-black) of the x-ray diffraction data for the powdered TaP crystal. The rows of ticks show the Bragg peak positions for the $I4_1md$ (space group No. 109), with a = b = 3.32 Å, and c = 11.34 Å.

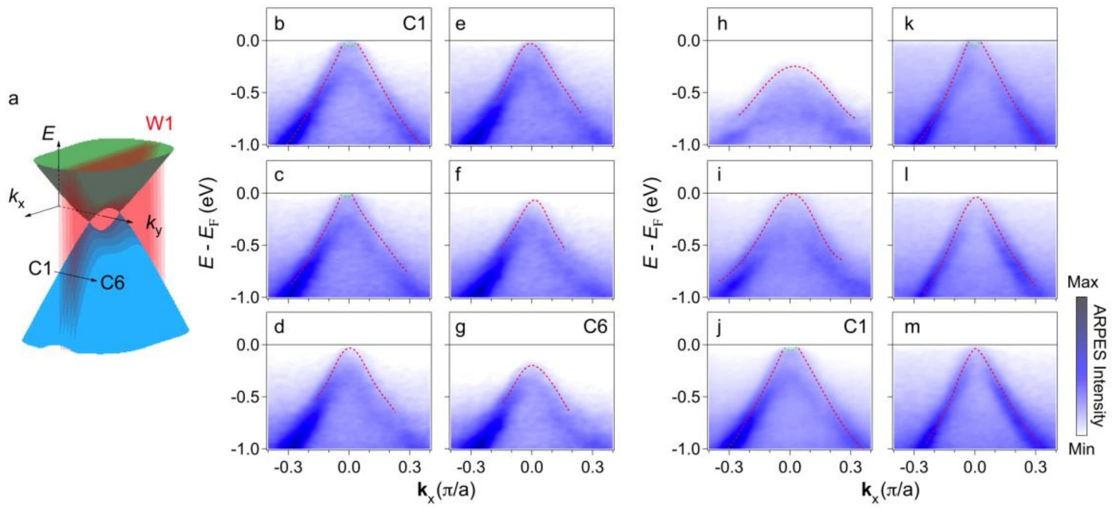

Supplementary Figure 2: Band evolution near the Weyl nodes W1. a, Schematic band dispersion of one pair of Weyl nodes in the $\mathbf{k}_x$-$\mathbf{k}_y$ plane. **b-g**, Band dispersions along the $\mathbf{k}_\mathrm{x}$ direction with $\mathbf{k}_\mathrm{z}$ = 20.58 π/c' and $\mathbf{k}_\mathrm{y}$ in a range of 0.54≥ $\mathbf{k}_\mathrm{y}$ ≥0.45 π/a. The cutting positions of C1-C6 are indicated by the vertical planes in **a**. **h-m**, Band dispersions along the $\mathbf{k}_\mathrm{x}$ direction with $\mathbf{k}_\mathrm{y}$ = 0.54 π/a and $\mathbf{k}_\mathrm{z}$ in a range of 20.3≤ $\mathbf{k}_\mathrm{z}$ ≤21.0 π/c'. The dotted lines in **b-m** are guides to the eye that indicate the band dispersions. For both in plane and out of plane direction, two bands cross each other twice at E_F (Weyl nodes) when cutting through two Weyl points perpendicular to the mirror plane (C1), and the two crossing points sink down and merge together while cutting off the Weyl points gradually. The results demonstrate that the Weyl nodes W1 form a pair of three-dimensional Dirac cone in the momentum space.

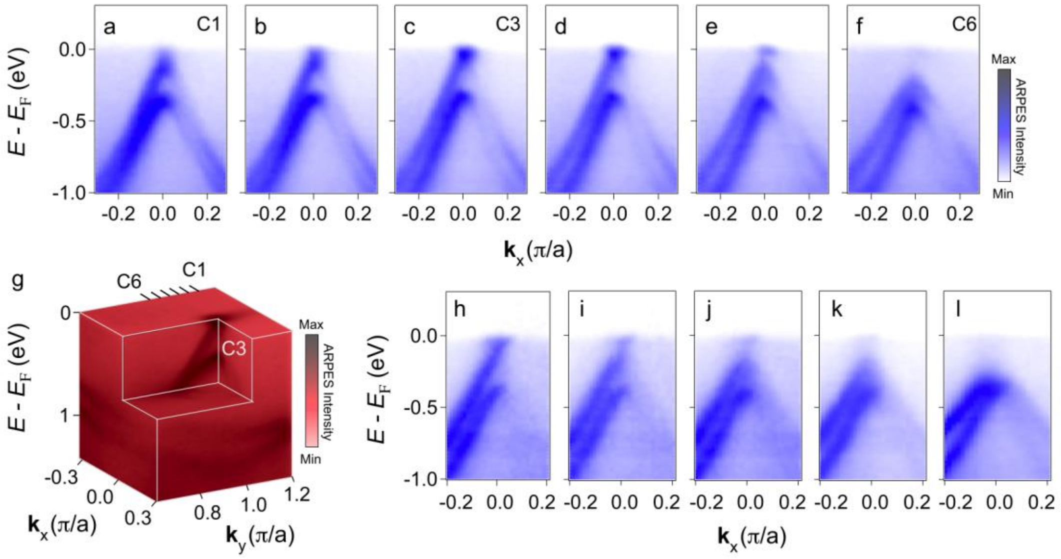

Supplementary Figure 3: Band evolution near the Weyl nodes W2. a-f, Band dispersions along the $\mathbf{k}_\mathrm{x}$ direction with $\mathbf{k}_\mathrm{z}$ = 20 π/c' and $\mathbf{k}_\mathrm{y}$ in a range of 1.13≥ $\mathbf{k}_\mathrm{y}$ ≥0.93 π/a. **g,** ARPES intensity profile near one pair of W2 in the $\mathbf{k}_x$-$\mathbf{k}_y$ plane. **h-l**, Band dispersions along the $\mathbf{k}_x$ direction with $\mathbf{k}_\mathrm{y}$ = 1.03 π/a and $\mathbf{k}_\mathrm{z}$ in a range of 21.0≤ $\mathbf{k}_\mathrm{z}$ ≤21.6 π/c'. The results indicate that W2 type of Weyl nodes form a pair of three-dimensional Dirac cone in the momentum space.

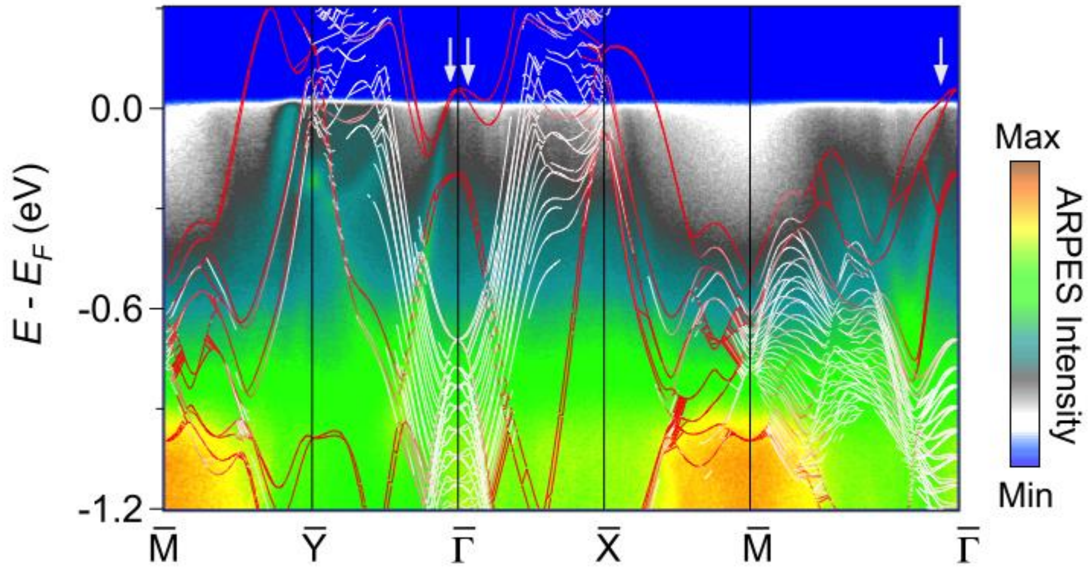

Supplementary Figure 4: Surface states on the P terminated (001) surface of TaP. ARPES spectra along high symmetry lines in surface BZ, compared with the calculated surface bands (red lines) on P-terminated (001) surface. The grey arrows indicate the surface states from P-terminated surface

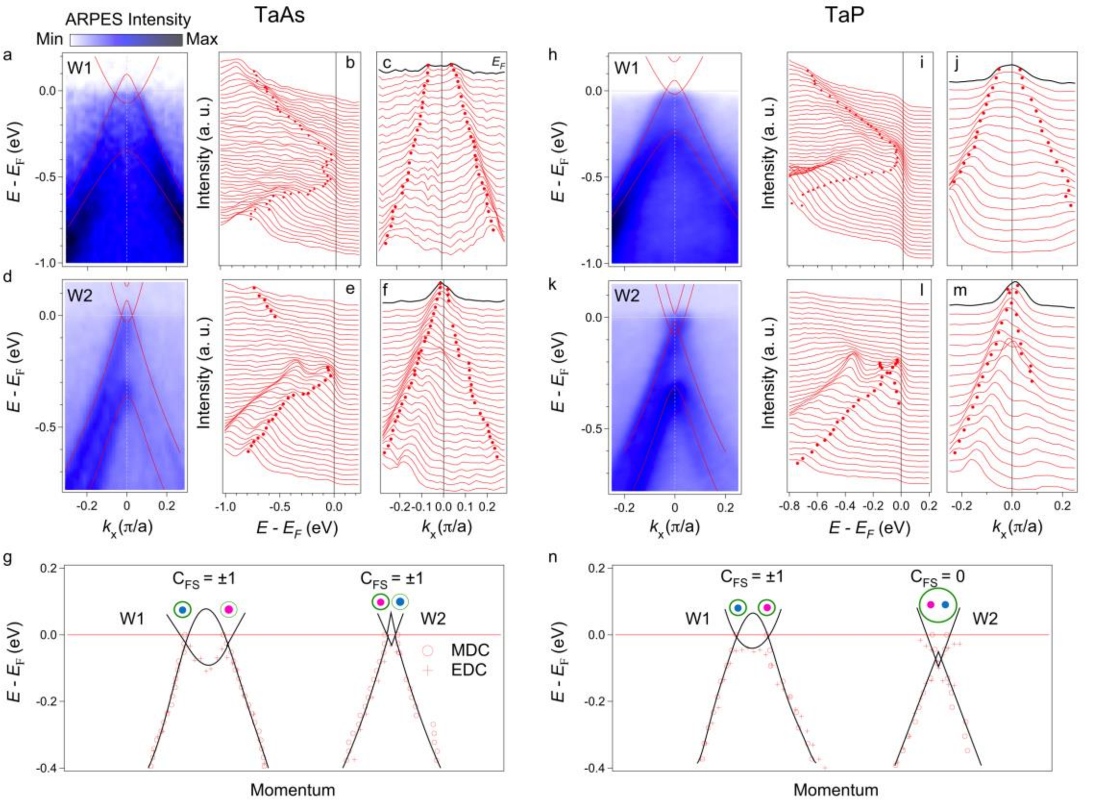

Supplementary Figure 5: Comparison of band structure near Weyl nodes between TaAs and TaP. a, ARPES spectra cutting through a pair of W1 in TaAs. Red lines are the band structure from calculations. **b-c,** Corresponding energy distribution curves and momentum distribution curves. Dots are the traced band dispersions. **d-f,** Same as **a-c**, but cutting through a pair of W2 in TaAs. **g,** Electronic band structure traced from experimental data. Each node of both W1 and W2 types in TaAs is enclosed by single Fermi surface with Fermi chern number $C_{FS} = \pm1$. **h-n,** Same as **a-g**, but for TaP. In TaAs every node of W1 and W2 types is enclosed by single Fermi surface with Fermi Chern number (C_{FS}) = ±1. In TaP the pair of W2 nodes are enclosed by one Fermi surface with zero C_{FS}, which is topological trivial. The data of TaAs was reproduced from previous work[1].

Supplementary Reference:

1. Lv B. Q., Xu N., Weng H. M., Ma J. Z., Richard P., Huang X. C., Zhao L. X., Chen G. F., Matt C., Bisti F., Strocov V. N., Mesot J., Fang Z., Dai X., Qian T., Shi M., and Ding H., *Observation of Weyl nodes in TaAs*, Nat. Phys. **11**, 724-727 (2015).
